# Veterinary access and communication barriers as predictors of owner stress among older adults

**DOI:** 10.3389/fvets.2026.1815971

**Published:** 2026-10-22

**Authors:** Rebecca M. Perley

**Affiliations:** Department of Health Care Management, California State University, Long Beach, CA, United States

**Keywords:** client stress, communication barriers, health system navigation, older adults, veterinary care access, veterinary workflow

## Abstract

**Background:**

Companion animal caregiving among adults aged 50 years and older is embedded within veterinary care systems, yet limited research has examined how perceived access and communication barriers influence owner wellbeing. Operational features such as appointment scheduling, staff accessibility, and communication clarity may function as stressors independent of pet illness severity.

**Methods:**

A total of 150 adults aged 50 years and older completed the survey. Valid sample sizes varied across descriptive variables because of item-level missingness, and the final hierarchical regression model included 133 participants with complete data on all model variables.

**Results:**

Veterinary access and communication barriers were significantly associated with higher owner stress (B = 0.32, SE = 0.10, β = 0.25, *p* = 0.002) after adjusting for gender identity, education, general health, frequency of veterinary visits, and pet health status. The full adjusted model explained 35.0% of the variance in owner stress (*R*^2^ = 0.35). Veterinary access and communication barriers explained an additional 5.1% of the variance beyond demographic and health-related factors and remained independently associated with higher owner stress. Poorer pet health was also associated with higher owner stress (β = 0.47, *p* < 0.001). However, the association between veterinary access and communication barriers and owner stress did not differ according to pet health status. Discussions of reduced-cost or pro bono services were not independently associated with owner stress.

**Conclusions:**

Perceived operational barriers within veterinary care systems represent meaningful contributors to owner stress among older adults. These findings highlight the importance of workflow design, communication clarity, and navigation supports as potential targets for improving client wellbeing in veterinary practice.

## Introduction

Companion animals play an important role in the lives of many older adults and community-dwelling individuals navigating later-life caregiving responsibilities. Veterinary care is therefore not only a clinical service, but a routine point of contact within broader caregiving environments. While research has documented the emotional benefits of pet ownership and the psychological impact of pet loss (1–3), comparatively less attention has been directed toward how veterinary care systems themselves shape owner wellbeing.

Access to care is widely recognized as a multidimensional construct that includes appointment availability, communication clarity, timeliness, and navigation processes. In human healthcare settings, navigation burden and fragmented communication systems have been linked to increased stress and reduced perceived control (4, 5). Similar dynamics may operate within veterinary contexts, where clients must coordinate logistics, interpret medical information on behalf of non-verbal patients, and implement treatment plans within their daily routines.

Emerging veterinary telehealth and telecommunication research further suggests that communication modality may influence both access to care and owner satisfaction. Studies examining virtual veterinary consultations and telehealth-supported management have demonstrated that remote communication approaches can improve accessibility while maintaining high levels of client satisfaction in both canine and feline care contexts (6, 7). These findings suggest that communication barriers may be shaped not only by interpersonal interactions, but also by the structural organization and delivery mode of veterinary services.

Veterinary research has demonstrated that client-veterinarian communication patterns influence client satisfaction, adherence to treatment recommendations, information exchange, and decision-making (8–11). In addition, studies examining veterinary care utilization have identified operational barriers—including appointment accessibility and perceived service complexity—as contributors to delayed or foregone care (12). These findings suggest that structural and communication-related processes within veterinary practice may function not only as determinants of whether and how clients access care, but also as sources of psychological strain. Communication barriers may include difficulty understanding veterinary terminology; challenges using digital communication systems; limited phone accessibility; accessibility barriers related to hearing or vision impairment, including difficulty hearing masked speakers or reading written instructions; uncertainty regarding next steps in care; or broader logistical and structural obstacles embedded within clinic workflows. Such barriers may operate across multiple levels, including the individual client level, clinic-level operational systems, and broader veterinary service environments.

These challenges may be particularly salient for community-dwelling older adults managing caregiving responsibilities within later-life health and caregiving contexts, although variation within this population warrants further investigation. Compared to younger populations, older individuals may experience fixed incomes, mobility limitations, and sensory or cognitive changes that heighten the complexity of navigating healthcare systems (5). When veterinary access processes are perceived as difficult or unclear, stress may emerge not solely from pet illness, but from the operational burden of coordinating care. Prior research has also demonstrated that caregiving burden among owners of ill companion animals may contribute substantially to emotional distress and perceived strain (13). These caregiving demands may intensify the psychological impact of communication and access barriers during periods of increased veterinary need.

Despite growing attention to financial barriers in veterinary care (12), less empirical work has examined how communication and scheduling barriers independently contribute to owner stress. Moreover, it remains unclear whether such barriers exert their influence primarily during periods of acute illness or whether they function as consistent stressors embedded within routine care delivery. The present study addresses this gap by examining whether perceived veterinary care access and communication barriers are associated with owner stress among adults aged 50 years and older, and whether that relationship varies according to pet health status. Using cross-sectional survey data, the study also assesses whether discussions of reduced-cost services independently influence stress.

Examining veterinary access among older adults also has broader relevance for gerontology and health services research because companion animal caregiving intersects with later-life health, caregiving responsibilities, resource navigation, and community-based living.

## Materials and methods

### Study design and participants

This study used a cross-sectional online survey design to examine associations between perceived veterinary care barriers and owner stress among adults aged 50 years and older. Participants were recruited through email communication channels associated with a university-affiliated lifelong learning institute at a large public university in the western United States. Recruitment materials included an invitation containing a link to an anonymous electronic survey.

OLLI is a community-based lifelong learning program designed for older adults and retirees, making it a practical setting for recruiting community-dwelling adults aged 50 years and older. Participation in OLLI is voluntary and includes individuals with diverse educational, occupational, and household backgrounds.

Although OLLI participants may not fully represent all older adult pet owners in the United States, the program provided access to a community-based population of older adults actively engaged in routine caregiving and healthcare decision-making contexts.

Eligibility criteria included being aged 50 years or older and currently owning or actively caring for a companion animal at the time of survey participation. Because survey questions focused on current veterinary experiences and perceptions of pet health, the study was designed to capture experiences that participants could reasonably recall and interpret within an active or recent caregiving context.

Participants were offered a $25.00 Amazon gift card incentive for survey completion through internal research funding support. A total of 150 respondents completed the survey. Because of item-level missingness, valid sample sizes varied across descriptive variables. The final hierarchical regression model included 133 participants with complete data on all variables included in the model.

### Measures

#### Owner stress and worry

Owner wellbeing was operationalized as frequency of stress and worry regarding pet health, reflecting the psychological burden associated with caregiving uncertainty and health-related decision-making. This construct was assessed using a single item: “How often do you feel stressed or worried about your pet's health?” Responses were measured on a 5-point Likert-type scale ranging from 1 (never) to 5 (very often), with higher scores reflecting greater stress.

#### Veterinary care barriers

Perceived veterinary care barriers were conceptualized as operational and communication-related challenges encountered when accessing veterinary care. The Veterinary Care Barriers composite consisted of four items assessing appointment access and communication accessibility (Q18, Q23, Q24, and Q25). Specifically, participants reported perceived difficulty obtaining appointments when needed, difficulty speaking with the veterinarian by phone, difficulty contacting veterinary assistants for care-related questions, and difficulty making appointments.

Q18 used a 5-point ordinal response scale originally coded from “extremely difficult” to “extremely easy” and was reverse-coded so that higher values reflected greater perceived difficulty. Q23–Q25 used ordinal response options (“No,” “Sometimes,” and “Yes”) that were coded numerically and directionally harmonized such that higher scores consistently reflected greater perceived communication or access difficulty.

Although response anchors differed slightly across items, all four items assessed conceptually related dimensions of operational access and communication barriers within veterinary care settings. Consistent with exploratory health services research approaches and early-stage scale development practices (14), the four items were averaged to create a composite Veterinary Care Barriers score, with higher scores indicating greater perceived access difficulty.

The reduced-cost/pro bono services item (Q50) was analyzed separately and was not included in the composite measure due to conceptual and psychometric differences.

Although the composite measure was not derived from a previously validated veterinary access instrument, items were developed to capture commonly reported operational and communication-related barriers identified in prior veterinary access and client communication literature (8, 9, 11, 12). Internal consistency estimates suggested acceptable reliability for exploratory use within this study.

#### Pet health status

Pet health status was assessed as a proxy for caregiving demand and perceived illness severity. Participants rated their pet's current health status using a single item: “How would you rate your pet's current health status?” Response options ranged from excellent to terrible on a 5-point scale, with higher scores indicating poorer perceived health.

#### Covariates

Gender identity was recoded as a binary dummy variable for regression analyses, with men coded as 0 and women coded as 1. Participants who selected “prefer not to say” or who had missing gender identity data were excluded from regression models through listwise deletion. Education level, general health rating, frequency of veterinary visits, and pet health status were entered as ordinal covariates. The final regression analytic sample included 133 participants with complete data on all variables included in the hierarchical regression model.

#### Survey items

Survey items were developed to align with the study objectives and reviewed for clarity and readability prior to distribution. The questionnaire was designed to capture practical experiences related to veterinary access, communication, and caregiving burden among older adults.

#### Scale construction and internal consistency

Veterinary Care Barriers were operationalized using a four-item composite index assessing perceived operational and communication-related barriers within veterinary care systems. Items included perceived difficulty obtaining appointments when needed, difficulty contacting the veterinarian by phone, difficulty contacting veterinary assistants for care-related questions, and difficulty making appointments. The scheduling item was reverse-coded so that higher scores consistently reflected greater perceived barriers. Items were averaged to create a composite Veterinary Care Barriers index (Cronbach's α = 0.77).

The primary outcome variable, owner stress and worry regarding pet health, was assessed separately using a single-item measure and was not included in the composite construction procedures. The reduced-cost/pro bono services item (Q50) was also analyzed separately due to conceptual differences from the operational access and communication items.

The composite measure was developed a priori for exploratory analytic purposes to capture theoretically related dimensions of veterinary care access within a cross-sectional framework. Full item wording and response options are provided in Appendix A.

#### Analytic strategy

Descriptive statistics were computed to characterize the sample and key study variables. Pearson correlations examined bivariate associations among owner stress, veterinary care barriers, pet health status, and covariates. Hierarchical linear regression models were then used to assess whether veterinary care barriers predicted owner stress after adjusting for theoretically relevant covariates and pet health status. The primary outcome variable across models was owner stress and worry regarding pet health.

For regression analyses, gender identity was recoded as a binary dummy variable, with men coded as 0 and women coded as 1. Participants who selected “prefer not to say” or who had missing gender identity data were excluded from regression models through listwise deletion. Education level, general health rating, frequency of veterinary visits, and pet health status were entered as ordinal covariates. In Step 1, gender identity, education level, general health rating, frequency of veterinary visits, and pet health status were entered simultaneously. In Step 2, the Veterinary Care Barriers composite score was added to assess incremental variance explained.

To examine whether the association between barriers and stress varied according to perceived pet health status, centered variables were used to construct an interaction term (veterinary care barriers × pet health status). This interaction term was entered in a subsequent moderation model. Additional regression models examined whether discussion of reduced-cost or pro bono veterinary services independently predicted owner stress when entered alongside veterinary care barriers.

Regression assumptions were evaluated prior to interpretation of results. Multicollinearity was assessed using tolerance and variance inflation factor (VIF) values. Residual normality was evaluated using a histogram and normal probability plot of standardized residuals, and the residual scatterplot was examined to assess the distribution of residuals across predicted values.

All analyses were conducted using IBM SPSS Statistics Version 29 (IBM Corp., Armonk, NY, USA). Statistical significance was set at *p* < 0.05.

The full survey questionnaire is provided in Appendix A to improve transparency and reproducibility of measurement procedures. Question identifiers were added to Appendix A to improve traceability between survey items and analytic variables. Question numbering reflects the original Qualtrics/SPSS variable structure; some questionnaire items, including open-ended questions and items not used in the final analyses, were omitted from the analytic dataset. Appendix B provides detailed coding and construction information for the Veterinary Care Barriers composite.

## Results

### Sample characteristics

The hierarchical regression analyses included 133 participants with complete data on all variables included in the final model. A total of 150 adults aged 50 years and older completed the survey, with valid sample sizes varying across descriptive variables because of item-level missingness. Participants had a mean age of 71.05 years (SD = 7.89), based on the 121 participants with valid age data. Among participants included in the final regression sample, reported stress regarding pet health was moderate (M = 2.81, SD = 0.81), and perceived veterinary care barriers were generally low to moderate (M = 1.63, SD = 0.62). Descriptive statistics for key study variables are presented in Table 1.

**Table 1:** Sample characteristics of participants.

Variable	*N*/Mean	%/(SD)
Demographic characteristics		
Age (years)	71.05	7.89
Gender identity		
Men	17	11.5%
Women	128	86.5%
Prefer not to say	3	2.0%
Education level		
High school graduate	2	1.4%
Some college	5	3.4%
Associate degree	12	8.1%
Bachelor's degree	62	41.9%
Professional degree	59	39.9%
Doctorate	8	5.4%
Living arrangement		
Live alone	52	35.4%
Live with spouse/partner	67	45.6%
Live with family	26	17.7%
Live with friends/roommates	2	1.4%
General health rating		
Excellent	52	35.1%
Good	74	50.0%
Average	15	10.1%
Poor	7	4.7%
Veterinary care and pet characteristics		
Frequency of veterinary visits		
More than twice a year	42	28.6%
Twice a year	36	24.5%
Once a year	38	25.9%
Less than once a year	16	10.9%
Only for emergencies	15	10.2%
Pet health status (M, SD)	1.95	0.88
Excellent	48	32.9%
Good	68	46.6%
Average	22	15.1%
Poor	6	4.1%
Terrible	2	1.4%
Key study variables in final regression sample (*N* = 133)		
Owner stress	2.81	0.81
Veterinary care barriers	1.63	0.62

Percentages are based on valid responses for each variable. Because of item-level missingness, the number of valid responses varies across variables. Descriptive statistics for owner stress and veterinary care barriers are reported for the final regression sample (*N* = 133). Age was available for a subset of participants (*n* = 121) because of missing or invalid responses.

### Bivariate associations

Veterinary care barriers were significantly correlated with owner stress (*r* = 0.31, *p* < 0.001), indicating that greater perceived difficulty with scheduling and communication was associated with higher levels of stress. Pearson correlations among study variables are shown in Table 2. Covariates were included based on theoretical relevance and prior literature rather than solely on bivariate associations.

**Table 2:** Pearson correlations among study variables.

Variable	1	2	3	4	5	6	7	
1. Owner stress	—							
2. Veterinary care barriers	0.31^***^	—						
3. Pet health status	0.51^***^	0.23^**^	—					
4. Gender identity: woman	−0.03	0.12	0.00	—				
5. Education level	−0.04	−0.04	0.08	0.06	—			
6. General health rating	0.20^*^	0.35^***^	0.31^***^	0.11	−0.12	—		
7. Frequency of veterinary visits	−0.13	0.21^*^	0.04	0.01	−0.09	0.18^*^	—	

Gender identity was dummy-coded for analysis, with men coded as 0 and women coded as 1. Correlations are based on participants with complete data on variables included in the final regression model (*N* = 133).

^*^p < 0.05; ^**^p < 0.01; ^***^p < 0.001.

### Hierarchical regression

In Step 1, gender identity, education level, general health rating, frequency of veterinary visits, and pet health status were entered simultaneously. The Step 1 model was statistically significant, *R*^2^ = 0.298, *F*_(5, 127)_ = 10.81, *p* < 0.001. Within this model, pet health status was the strongest predictor of owner stress (β = 0.505, *p* < 0.001), indicating that poorer perceived pet health was associated with higher owner stress. Frequency of veterinary visits was also significantly associated with owner stress (β = −0.165, *p* = 0.031).

In Step 2, Veterinary Care Barriers were added to the model and accounted for a significant additional 5.1% of the variance in owner stress, Δ*R*^2^ = 0.051, Δ*F*(1, 126) = 9.92, *p* = 0.002. The final model was statistically significant, *R*^2^ = 0.350, *F*_(6, 126)_ = 11.29, *p* < 0.001. In the final model, Veterinary Care Barriers remained a significant independent predictor of owner stress (B = 0.32, SE = 0.10, β = 0.248, *p* = 0.002), controlling for gender identity, education level, general health rating, frequency of veterinary visits, and pet health status. Poorer pet health status also remained significantly associated with higher owner stress (β = 0.470, *p* < 0.001), as did frequency of veterinary visits (β = −0.203, *p* = 0.007). Gender identity, education level, and general health rating were not statistically significant predictors of owner stress in the final model. Full regression results are presented in Table 3.

**Table 3:** Hierarchical linear regression predicting owner stress (*N* = 133).

Predictor	Step 1 β	Step 2 β
Gender identity: woman	−0.029	−0.051
Education level	−0.082	−0.080
General health rating	0.070	0.004
Frequency of veterinary visits	−0.165^*^	−0.203^**^
Pet health status	0.505^***^	0.470^***^
Veterinary care barriers	—	0.248^**^

Model statistics. Step 1: R^2^ = 0.298, F_(5, 127)_ = 10.81, *p* < 0.001.

Step 2: R^2^ = 0.350, ΔR^2^ = 0.051, Δ*F*(1,126) = 9.92, *p* = 0.002.

Final Model: F_(6, 126)_ = 11.29, *p* < 0.001.

^*^p < 0.05; ^**^p < 0.01; ^***^p < 0.001.

Model diagnostics indicated no evidence of problematic multicollinearity, with tolerance values ranging from 0.792 to 0.973 and VIF values ranging from 1.028 to 1.263 in the final model. The Durbin-Watson statistic was 2.03. Visual inspection of the residual histogram and normal probability plot indicated that standardized residuals were approximately normally distributed. The residual scatterplot did not indicate substantial deviation from regression assumptions.

### Financial mitigation

When discussion of reduced-cost or pro bono services was entered alongside veterinary care barriers, it was not a significant predictor of stress (β = 0.10, *p* = 0.228), and did not meaningfully increase explained variance.

### Moderation analysis

To test whether the relationship between veterinary care barriers and owner stress differed according to pet health status, an interaction term combining veterinary care barriers and pet health status was added to a separate regression model after including the individual effects of both variables. The interaction was not statistically significant (β = 0.03, *p* = 0.72), indicating that the association between veterinary care barriers and owner stress did not vary according to perceived pet health status.

## Discussion

### Overview of key findings

This study examined whether perceived veterinary care access and communication barriers were associated with owner stress among adults aged 50 years and older. Findings indicate that veterinary access barriers remained a significant independent predictor of owner stress after adjusting for demographic characteristics, general health, frequency of veterinary visits, and pet health status. Pet health status was the strongest predictor in the final model, indicating that poorer perceived pet health was associated with higher owner stress. However, veterinary care barriers contributed additional explanatory value beyond pet health and covariates, suggesting that operational and communication-related barriers represent a distinct source of owner stress within veterinary care contexts. Discussions of reduced-cost or pro bono services were also not independently associated with owner stress when considered alongside veterinary access and communication barriers.

### Barriers as system-level stressors

The findings of this study suggest that operational features of veterinary practice—rather than clinical severity alone—may meaningfully shape client psychological experiences. Difficulty scheduling appointments, reaching veterinary staff by phone, or navigating communication processes can introduce uncertainty and anticipatory stress. These operational frictions may increase uncertainty, reduce confidence in navigating care, and contribute to psychological strain, particularly among older adults managing caregiving responsibilities within broader health and logistical contexts.

In human healthcare research, navigation burden and fragmented communication systems have been associated with increased stress, delayed care engagement, and reduced perceived control (4, 5). Similar dynamics may operate within veterinary settings, where clients coordinate logistics, interpret medical information, and implement treatment recommendations on behalf of non-verbal patients. When communication systems are perceived as difficult to navigate or insufficiently responsive, stress may emerge independently of illness severity itself.

The absence of moderation by pet health severity further underscores this interpretation. Barriers were associated with stress regardless of whether pets were perceived as being in excellent or poor health. This pattern suggests that structural and process-related friction functions as a consistent stressor, not merely as a reaction to crisis-level illness. Even routine care encounters may generate strain when operational systems create obstacles to timely communication or appointment coordination.

These findings position veterinary access systems as psychosocial environments that influence client wellbeing. While financial constraints remain important considerations in veterinary care, the present results indicate that operational accessibility and communication clarity may exert independent and measurable effects on client stress.

### Why pet health did not moderate the effect

The study hypothesized that veterinary care barriers would exert a stronger effect on stress among those caring for pets in poorer health; however, this interaction was not statistically significant. Perceived pet health status did not change the strength of the relationship between veterinary access barriers and owner stress. The association between barriers and stress remained consistent across levels of perceived illness severity. This finding suggests that operational and communication-related friction functions as a stable stressor rather than one that intensifies only during periods of medical crisis.

The finding that pet health status did not change the strength of the association between veterinary access and communication barriers and owner stress also highlights the distinction between medical acuity and system design. Structural barriers appear to contribute to owner stress across different levels of pet illness severity, suggesting that difficulties with access and communication are distinct sources of stress rather than merely consequences of caring for a sicker pet.

One possible explanation is that limited variability in perceived veterinary care barriers, pet health status, or owner stress may have reduced the ability to detect whether the association between barriers and stress differed according to pet health status.

### Financial mitigation is not the same as access

An important and perhaps counterintuitive finding was that whether a veterinarian had offered or discussed reduced-cost or pro bono services was not independently associated with owner stress when considered alongside access and communication barriers. While financial constraints are frequently cited as central obstacles in veterinary care, these results suggest that financial mitigation discussions alone may not sufficiently reduce psychological strain if operational and communication barriers remain unresolved.

One possible explanation is that financial assistance discussions may address a specific transactional barrier without resolving broader uncertainty related to scheduling, communication, follow-up care, or treatment implementation.

Additionally, financial mitigation does not directly address difficulties in communication, follow-up, or other aspects of the clinic experience. Difficulty reaching staff, delays in appointment availability, or unclear communication channels may generate anticipatory stress independent of cost considerations. The present findings suggest that while affordability remains a critical dimension of veterinary access, it should be conceptualized as one component of a broader access ecosystem. Structural accessibility and communication clarity may be equally—if not more—salient contributors to client wellbeing.

These results underscore the importance of distinguishing between financial barriers and operational access barriers when designing interventions aimed at reducing client stress. Furthermore, these findings are consistent with prior veterinary access research demonstrating that financial cost represents only one dimension of care accessibility and decision-making complexity (12).

### Implications for veterinary practice and clinic operations

The findings of this study carry direct implications for veterinary clinic operations and client experience design. If perceived barriers to scheduling, communication, and appointment access are independently associated with elevated client stress, then operational structures within veterinary practices represent modifiable determinants of client wellbeing.

Improving appointment accessibility may involve reducing barriers to scheduling, such as long telephone hold times, limited appointment options, and difficulty accessing a preferred veterinarian, alongside more consistent callback protocols and mechanisms for triaging non-urgent inquiries. Targeted workflow improvements—such as training front-desk staff to document client concerns accurately and communicate them clearly to the appropriate veterinary team member, establishing clear protocols for identifying messages that require timely clinical review, and providing individualized after-visit summaries and treatment plans tailored to the animal's condition and the client's ability to understand and carry out the recommended care—may reduce client uncertainty about next steps and treatment implementation, as well as anticipatory stress. Practices may also benefit from examining how communication channels are organized, particularly for older clients who may prefer phone-based contact but encounter extended hold times or receive incomplete or inconsistent information from different members of the veterinary team. Practices should also consider accessibility needs among clients with hearing or vision impairments by offering communication in multiple formats, such as written and verbal instructions, large-print materials, captioned or text-based communication, and opportunities to confirm understanding.

Importantly, these findings suggest that client stress is not solely a function of medical complexity. Even when pets are perceived as being in good health, operational friction may contribute to client psychological strain. This highlights the importance of treating client experience as an integral component of veterinary care delivery rather than as a peripheral concern. Many veterinary practices have intentionally implemented communication, scheduling, documentation, and follow-up procedures to support clients. However, the effectiveness of these processes may depend on how consistently they are implemented, how well information is transferred across team members, and whether they are responsive to clients with differing communication and accessibility needs. Clear communication, accurate documentation of client concerns, timely escalation of clinically relevant messages, reliable scheduling processes, and consistent follow-up may strengthen trust, support continuity of care, and improve clients' confidence in the practice. Over time, these processes may also influence treatment adherence and clients' decisions to remain with a veterinary practice, with potential implications for practice sustainability. Prior veterinary communication research has similarly demonstrated that communication clarity and continuity contribute to client satisfaction, trust, and adherence to treatment recommendations (9, 11).

While communication training for veterinary teams remains an important consideration, the present results suggest that structural workflow design deserves equal attention. Practices may support treatment implementation by providing individualized after-visit materials, including clear discharge instructions and step-by-step guidance for carrying out the treatment plan, with opportunities for clients to review the plan and confirm understanding. Consistent follow-up communication may further reduce uncertainty and improve confidence in treatment implementation. Emerging digital communication tools and follow-up systems may offer practical ways to improve care navigation—for example, by providing plain-language visit summaries, outlining future care options and anticipated timelines, such as considering surgery within the next two months, sending reminders about medications or follow-up appointments, and offering clear guidance about whom to contact and what information to bring when seeking care at an emergency animal hospital outside normal clinic hours.

Taken together, these findings encourage veterinary practices to consider how operational systems—beyond clinical expertise alone—shape client stress, satisfaction, and engagement with care.

### Conceptual contribution

Beyond its operational implications, this study contributes to multiple intersecting bodies of scholarship by conceptualizing veterinary care systems as structural determinants of client wellbeing. While research on companion animals has traditionally emphasized the psychological benefits of pet ownership or the emotional consequences of pet loss, comparatively little attention has been directed toward how veterinary care processes themselves shape owner stress. By isolating access and communication barriers as independent predictors of stress, this study shifts the analytic focus from pet illness alone to the systems through which care is delivered.

These findings align with and extend health services research demonstrating that navigation burden and communication friction function as stress pathways within human healthcare settings (4, 5). The present study suggests that similar mechanisms operate in veterinary contexts, where clients serve as proxies for non-verbal patients and assume primary responsibility for treatment implementation. In this way, veterinary systems become embedded within broader caregiving environments, particularly for older adults.

The finding that pet health status did not change the strength of the relationship between veterinary access and communication barriers and owner stress further strengthens this interpretation. Structural barriers appear to contribute to owner stress across different levels of pet illness severity, suggesting that difficulties with access and communication are distinct sources of stress rather than merely consequences of caring for a sicker pet.

By integrating insights from veterinary medicine, health system navigation research, and aging studies, this work advances an interdisciplinary understanding of how companion animal care intersects with broader caregiving and autonomy contexts. It positions veterinary access not merely as a matter of service delivery, but as a component of the caregiving infrastructure surrounding older adults.

## Limitations

Several limitations should be considered when interpreting these findings.

First, the cross-sectional design precludes causal inference. While veterinary access barriers were strongly associated with owner stress, the directionality of this relationship cannot be definitively established. It is possible that individuals experiencing higher stress may perceive access processes as more burdensome. Longitudinal research is needed to clarify temporal sequencing.

Second, the sample was relatively homogeneous in age distribution, which may limit detection of age-related differences.

Third, measures relied on self-report. Perceived barriers and stress may differ from objective clinic measures, such as the number of days until an appointment is available, the time required for the clinic to return calls or messages, or whether a pet's condition requires urgent, extended, or specialized care. However, perceived barriers are themselves meaningful, as stress responses are shaped by clients' experiences of access and communication rather than by objective clinic measures alone.

Fourth, several study variables—including perceived pet health status, participants' self-rated general health, and the Veterinary Care Barriers composite—were measured using self-reported items that had not undergone formal external validation. The Veterinary Care Barriers composite combined four related but not identical items assessing difficulty obtaining appointments, reaching the veterinarian by phone, contacting veterinary assistants with care-related questions, and making appointments. Although the composite demonstrated acceptable internal consistency for exploratory analysis, the use of items with differing numbers of response categories may have reduced measurement precision and may not have captured important distinctions among scheduling access, communication with the veterinarian, and communication with other clinic staff.

Fifth, the sample consisted of adults affiliated with a lifelong learning institute and may not reflect the experiences of the broader population of older pet owners. This affiliation may indicate greater engagement in educational and community activities than is typical of some segments of the older adult population, which may limit generalizability to individuals with different socioeconomic circumstances, health needs, levels of social engagement, or patterns of veterinary care use. Future research should examine more diverse samples to determine whether veterinary access and communication barriers operate similarly across populations with differing resources and care contexts.

Sixth, voluntary recruitment through a lifelong learning institute may have introduced selection bias. Individuals who participate in community educational programming and choose to complete a survey about companion animals may differ from the broader population of older adult pet owners, particularly in their level of engagement with veterinary care and companion animal caregiving.

Seventh, although the sample size was sufficient to detect several statistically significant associations, larger and more geographically diverse samples would permit more precise evaluation of subgroup differences and improve generalizability.

Eighth, the financial mitigation measure did not capture the full range of financial strain, insurance coverage, or cost-related veterinary care decisions. More detailed assessment could clarify how affordability influences choices such as purchasing prescribed medications, consenting to overnight observation, pursuing diagnostic testing, or selecting among treatment options. Although financial costs did not explain owner stress beyond veterinary practice barriers in the present analysis, they may still shape specific care decisions and pet health outcomes.

Ninth, age was described as a sample characteristic, but the final regression models did not examine differences across age subgroups or test age as a moderator. Therefore, the study cannot determine whether the observed associations differ by age.

Despite these limitations, the strength and consistency of the observed associations suggest that operational access barriers represent a meaningful and measurable contributor to client stress within veterinary care contexts.

## Future directions

Future research should extend these findings through longitudinal designs that examine how access barriers influence stress trajectories over time, particularly during transitions such as diagnosis, treatment escalation, or end-of-life decision-making. Additional qualitative and mixed-methods research may help clarify the mechanisms through which communication and access barriers contribute to owner stress among older adults. Such work may inform future experimental or quasi-experimental studies evaluating workflow modifications, structured follow-up protocols, or enhanced communication systems designed to reduce operational friction and improve client wellbeing.

Qualitative approaches such as focus groups or in-depth interviews may further illuminate how older adults understand explanations of their animal's condition, anticipated course of care, available treatment options, and the likely consequences of pursuing or declining particular interventions. Such approaches could also clarify how clients interpret uncertainty, ask follow-up questions, navigate communication barriers, and experience stress related to companion animal caregiving.

Additionally, the development and evaluation of digital navigation supports represent a promising avenue for innovation. AI-assisted visit summaries, medication and follow-up reminders, plain-language explanations of treatment options, and guidance about whom to contact when questions or urgent concerns arise may help clients better understand and carry out veterinary care plans. These tools should support, rather than replace, communication with the veterinarian and other members of the veterinary team. Rigorous pilot testing in clinical settings will be necessary to assess feasibility; acceptability to clients, veterinarians, and clinic staff; and effects on client stress, veterinarian and staff workload, mutual understanding between clients and the veterinary team, and clinic efficiency.

Finally, interdisciplinary collaboration among veterinary professionals, geriatricians, social workers, other healthcare providers serving older adults, and health systems researchers may support more integrated approaches to companion animal caregiving in later life. Understanding how veterinary access intersects with older adults' physical and mental health, companion animal health, caregiving responsibilities, resource availability, and community-based living contexts represents an important next step in advancing both client-centered veterinary practice and holistic aging research.

## Conclusion

Difficulty obtaining an appointment when needed and completing the appointment-scheduling process itself, challenges reaching veterinarians by phone, and difficulty contacting veterinary assistants with care-related questions represent meaningful and measurable contributors to owner stress among older adults. Independent of pet health severity, friction within scheduling and communication systems appears to function as a structural stressor that increases uncertainty, complicates owners' efforts to obtain veterinary care for their companion animals, and adds to the psychological burden of caring for a companion animal. Addressing these barriers through workflow refinement, clearer communication processes, and navigation supports may improve the client experience, support more consistent implementation of care plans, lessen psychological strain, and contribute to better health outcomes for companion animals. Recognizing veterinary access systems as components of broader caregiving environments may help inform future efforts to improve communication, accessibility, and well-being among older adult pet owners, who often serve as the primary advocates and decision-makers for animals that cannot communicate their needs directly.

## Data Availability

The raw data supporting the conclusions of this article are not publicly available because the informed consent and confidentiality provisions for this study restrict access to authorized study personnel. Requests for additional information may be directed to the corresponding author.
