# Supplementary material for: Veterinary access and communication barriers as predictors of owner stress among older adults
Source: Front Vet Sci. 2026 Oct 22;13:1815971. doi: 10.3389/fvets.2026.1815971 (PMC13736126; doi:10.3389/fvets.2026.1815971)
Supplement: Supplementary file 1 [file Data_Sheet_1.pdf]

Supplementary Material

Veterinary access and communication barriers as predictors of owner stress among older adults

Rebecca M. Perley

This file contains the survey questionnaire and the coding and construction details for the Veterinary Care Barriers composite referenced in the article.

Supplementary Appendix A. Survey Questionnaire

Section 1: Sociodemographic Information

- Q5. Year of Birth (please enter the year you were born)
- Q6. Gender (Male, Female, Non-binary/third gender, Prefer not to say)
- Q7. Living arrangement (Live alone, Live with spouse/partner, Live with family, Live with friends/roommates, Other)
- Q8. Employment status (Employed full-time, Employed part-time, Retired, Unemployed, Homemaker / Caregiver, Other)
- Q9. Education level (Less than high school, High school graduate, Some college, 2 year / Associate degree, 4 year / Bachelor's degree, Professional degree, Doctorate)
- Q10. General health rating (Excellent, Good, Average, Poor, Terrible)

Section 2: Pet Ownership

- Q11. Type(s) of pet(s) owned (Dog, Cat, Bird, Small mammal (e.g., rabbit, guinea pig), Reptile / amphibian, Other (please specify)
- Q12. Length of ownership (years) (How many years have you owned your current pet(s)?)
- Q13. Primary caregiver status (I am the primary caregiver, I share caregiving responsibilities, Someone else in the household is the primary caregiver)
- Q14. Frequency of veterinary visits (More than twice a year, Twice a year, Once a year, Less than once a year, Only for emergencies)
- Q15. Pet's current health status (Excellent, Good, Average, Poor, Terrible)

Section 3: Barriers to Veterinary Care

- Q16. In the past 12 months, have you experienced difficulty obtaining veterinary care? (Yes / No)
- Q17_1 to Q17_6. If yes, what were the reasons? (Cost, Transportation, Availability (difficulty getting appointments), Communication with staff, Mobility or health limitations, Other (please specify)
- Q18. How easy is it for you to schedule a veterinary appointment when needed? (Extremely difficult, Somewhat difficult, Neither easy nor difficult, Somewhat easy, Extremely easy)
- Q19. How would you rate your level of trust in your veterinarian? (Very high, High, Moderate, Low, Very low)
- Q20. If your veterinarian recommended a high-cost procedure (for example, a CT scan costing around $7,500), do you have someone who could help you organize or finance this care? (Yes / Maybe / No)
- Q21. What would you most likely do if you could not afford a recommended treatment for your pet? (Delay care, Seek a lower-cost option, Decline treatment and provide comfort care, Consider euthanasia, Other – please describe)
- Q22. Have you ever delayed or avoided seeking veterinary care due to cost or other barriers? (Yes, No)

Section 4: Veterinarian–Owner Communication and Accessibility

- Q23. Is your veterinarian hard to talk to on the phone? (Yes, No, Sometimes)
- Q24. Is it hard to contact your veterinarian's assistant for care questions? (Yes, No, Sometimes)
- Q25. Is it hard to make an appointment with your veterinarian? (Yes, No, Sometimes)
- Q26. Does your veterinarian take the time to do a complete physical examination (for example, removing a harness or shirt to view your pet's body)? (Strongly agree, Somewhat agree, Neither agree nor disagree, Somewhat disagree, Strongly disagree)

- Q27. Does your veterinarian confirm instructions for all medications being given, even when only one is being changed? (Definitely not, Probably not, Might or might not, Probably yes, Definitely yes)
- Q28. Are you notified of potential next steps when discussing your pet's chronic or acute illness (for example, possible CT scan, internist consultation, or referral)? (Strongly agree, Somewhat agree, Neither agree nor disagree, Somewhat disagree, Strongly disagree)
- Q29. Does your veterinarian proactively discuss euthanasia and quality of life with you when dealing with your pet's chronic health condition? (Definitely not, Probably not, Might or might not, Probably yes, Definitely yes, Not applicable)
- Q30. When your pet's condition became critical, did your veterinarian prepare you for the possibility that your pet might pass away at home? (Yes / No / Not applicable)
- Q31. If yes, were you informed about what to do next? (Yes, No)
- Q32. Did your veterinarian inform you that other conditions or complications could arise with your pet's illness or treatment (for example, opportunistic diseases or side effects)? (Strongly agree, Somewhat agree, Neither agree nor disagree, Somewhat disagree, Strongly disagree, Not applicable)
- Q33. When a pet or a person in your household passed away, did your veterinarian or another professional provide any guidance or support for helping your remaining pet(s) cope with the loss? (Yes / No / Not applicable)
- If no, what kind of information or support would have been helpful for you and your remaining pet(s)? (Open-ended)
- Q38. Have you ever sought a second opinion from another veterinarian? If yes, what was the reason? (Yes, No)
- Q39. If yes, what was the reason? (text-entry question)

Section 5: Owner Well-Being

- Q40. How often do you feel stressed or worried about your pet's health? (Never, Rarely, Sometimes, Often, Very often)
- Q41. How much does caring for your pet affect your physical health? (Not at all, A little, Somewhat, Quite a bit, Very much)
- Q42. How much does caring for your pet affect your emotional well-being? (Not at all, A little, Somewhat, Quite a bit, Very much)
- Q43. Since owning your pet, would you say your overall quality of life has: (Improved, Stayed the same, Declined)
- Q44. After a difficult veterinary visit, how long do you usually feel worried or unsettled? (Not at all, A few hours, A day, Several days, More than a week, Not applicable)
- Q45. How supported do you feel by your veterinarian when your pet's health declines? (Not at all supported, Slightly supported, Somewhat supported, Very supported, Extremely supported, Not applicable)
- Q46. Do veterinary experiences affect your sleep, appetite, or daily activities? (Never, Rarely, Sometimes, Often, Always)
- Q47. Do you ever feel uncertain or uninformed during veterinary discussions, leading you to follow recommendations without asking questions? (Never, Rarely, Sometimes, Often, Always)
- Q48. After a veterinary visit, do you often think of additional questions but find it difficult to contact the veterinarian or staff to get answers? (Never, Rarely, Sometimes, Often, Always)
- Q49. Have you ever felt guilt, sadness, or distress about being unable to afford recommended veterinary care for your pet? (Never, Rarely, Sometimes, Often, Always, Not applicable)
- Q50. Has your veterinarian ever offered or discussed reduced-cost or pro bono services for your pet's care? (Yes, No, Not sure)

- Q51. If yes, or if you wish such services had been available, please describe how this affected your experience with your veterinarian or your ability to care for your pet. (Open-ended)

Section 6: Technology and Pet Care Support

- Q48_0. Are you comfortable using artificial intelligence (AI) tools or online systems to help you understand how medication and medical care impact your pet? (Strongly agree, Agree, Neither agree nor disagree, Disagree, Strongly disagree)
- Q49_0. Would you be open to AI-based tools that help track your pet's health or explain veterinary recommendations? (Strongly agree, Agree, Neither agree nor disagree, Disagree, Strongly disagree)

Section 7: Open-Ended Questions

- Q50_0. What has been your most positive experience with your veterinarian?
- Q51_0. What has been your most challenging experience with your veterinarian?
- Q52. What changes would you like to see in veterinary care for older adults and their pets?

Supplementary Appendix B. Veterinary Care Barriers Composite Construction

Variable	Survey Item	Coding
Q18	Difficulty obtaining a veterinary appointment when needed	Originally coded from 1 = Extremely difficult to 5 = Extremely easy; reverse coded so higher values reflected greater perceived difficulty
Q23	Veterinarian hard to talk to on phone	1 = No, 2 = Sometimes, 3 = Yes
Q24	Difficulty contacting veterinarian's assistant	1 = No, 2 = Sometimes, 3 = Yes
Q25	Difficulty making veterinary appointment	1 = No, 2 = Sometimes, 3 = Yes

Note. All items were directionally harmonized such that higher scores reflected greater perceived operational access and communication difficulty prior to averaging into the Veterinary Care Barriers composite score.
